# Beyond the gut: spectrum of magnetic surgery devices

**DOI:** 10.3389/fsurg.2023.1253728

**Published:** 2023-10-24

**Authors:** William G. Lee, Lauren L. Evans, Michael R. Harrison

**Affiliations:** ^1^ Department of Surgery, Cedars-Sinai Medical Center, Los Angeles, CA, United States; ^2^ Department of Surgery, University of California San Francisco, San Francisco, CA, United States

**Keywords:** magnetic surgery, non-gastrointestinal, magnetic levitation, pectus excavatum, scoliosis, sleep apnea, vascular port anastomosis

## Abstract

Since the 1970s, magnetic force has been used to augment modern surgical techniques with the aims of minimizing surgical trauma and optimizing minimally-invasive systems. The majority of current clinical applications for magnetic surgery are largely centered around gastrointestinal uses—such as gastrointestinal or bilioenteric anastomosis creation, stricturoplasty, sphincter augmentation, and the guidance of nasoenteric feeding tubes. However, as the field of magnetic surgery continues to advance, the development and clinical implementation of magnetic devices has expanded to treat a variety of non-gastrointestinal disorders including musculoskeletal (pectus excavatum, scoliosis), respiratory (obstructive sleep apnea), cardiovascular (coronary artery stenosis, end-stage renal disease), and genitourinary (stricture, nephrolithiasis) conditions. The purpose of this review is to discuss the current state of innovative magnetic surgical devices under clinical investigation or commercially available for the treatment of non-gastrointestinal disorders.

## 1. Introduction

Magnetic surgical devices have been utilized to augment existing minimally-invasive surgical (MIS) techniques with the aims of minimizing surgical trauma and creating novel MIS approaches to address pathology in children and adults (1). As MIS approaches seek to achieve similar outcomes through the smallest incisions possible, post-operative morbidity, convalescence period, and overall cost can all be optimized (2). In addition, the combination of endoscopy with magnetic surgical approaches has allowed for the development of entirely endoscopic—or “incision-less” approaches—to further minimize surgical trauma. While development of these approaches remains under ongoing development, they have already demonstrated a clear benefit in patients with significant medical or anatomic comorbidities that may be prohibitive to conventional surgical approaches (3).

Since the use of electromagnetic-driven bougienage for esophageal lengthening in esophageal atresia by Dr. Hendren and Dr. Hale in 1975, early uses of magnetic force in surgery were focused on the gastrointestinal (GI) tract such as colostomy closure devices (4), bowel anastomosis ring devices (5), and biliary-enteric anastomosis devices (6). Extensive experience from the development of GI-focused devices led to the categorization of six core technical principles in magnetic surgery: compression, anchoring, levitation, tracing, navigation, and driving. Modern in-human use of magnetic force in GI surgery now utilizes these principles to guide device development. Magnetic compression is used to create devices which form new esophageal or bowel anastomoses (3, 7), as well as perform stricturoplasty in the GI or biliary tracts (8–10). Devices founded on magnetic anchoring have been developed to provide surgical retraction of intra-abdominal organs during laparoscopic cholecystectomy (11, 12) and augment bariatric procedures (13, 14), while magnetic navigation is integrated into a commonly used device to guide placement of modern nasoenteric feeding tubes (15).

The successful application of these core magnetic surgical principles in GI surgery has served as a template for the clinical application of magnetic force in other non-GI organ systems including the musculoskeletal, respiratory, cardiovascular, and genitourinary systems (Table 1). The Surgical Innovations group at the University of California San Francisco has been studying the use of magnetic force for unsolved surgical problems for more than two decades. Initially, the magnetic compression technique for GI anastomoses was developed in animal models (16–20), then clinically for esophageal atresia (3, 7), biliary-enteric anastomoses (21), and duodenal-ileal anastomoses to treat type two diabetes mellitus (22). Magnetic therapies were then developed for pectus deformities, spinal deformities, and then for obstructive sleep apnea (Table 1). Prior reviews have focused on the use of magnetic force for GI conditions, as well as the bioengineering principles behind magnetic device development (23). In this review, we concentrate on non-GI applications by many groups around the world. This review serves to provide a brief overview of the magnetic surgical devices beyond the GI tract, with a focus on devices approved for humanitarian or commercial in-human use. While this review does not cover all existing devices, the objective of this review is to discuss the adaptation of core magnetic surgical principles from existing GI-focused devices in the treatment of non-GI disorders.

**Table 1:** Published in-human magnetic surgical devices for non-gastrointestinal-related use.

Core technical principle	Device	Organ system	Indication	Function	Limitations
Magnetic driving	MAGEC System (NuVasive, Inc., San Diego, CA, USA)	Musculoskeletal	Scoliosis	Non-invasive dynamic adjustment of internal implanted spinal growth rods	Device failure/fracture, Infection, Cost
Magnetic driving	PRECICE Intramedullary Limb Lengthening System (NuVasive, Inc., San Diego, CA, USA)	Musculoskeletal	Limb length discrepancy, Congenital limb malformation	Non-invasive dynamic adjustment of implanted telescoping intramedullary nail	Discomfort, Joint stiffness/subluxation, Delayed union, Infection, Device fracture
Magnetic levitation	Magnetic Mini-Mover (3MP): Magnimplant and Magnatract (Hayes Manufacturing, Sunnyvale, CA and Hantel Technologies, Hayward, CA)	Musculoskeletal	Pectus excavatum	Gradual remodeling of congenital chest wall deformity	Discomfort, Titanium fixation cable fracture, Reliance on patient adherence to external brace, Delayed treatment response
Magnetic levitation	Magnetic Apnea Prevention Device (MAGNAP) (Magnap, Inc., UCSF Surgical Innovations, San Francisco, CA, USA)	Respiratory	Obstructive sleep apnea	Hyoid bone advancement during sleep to maintain upper airway patency	Reliance on patient adherence to external brace, Need for Phase I/II efficacy data
Magnetic anchor	Magnetic Blackstar (Urovision-Urotech, Achenmuhle, Germany)	Genitourinary	Procedures requiring ureteral stent placement	Bedside removal of ureteral stent without the need for cystoscopy	Discomfort, Lack of procedural anesthesia, Difficulty with stent insertion and removal
Magnetic compression	Magnetic Vascular Positioner (MVP) Series 6,000 Distal Anastomosis System (Ventrica, Inc., Fremont, CA, USA)	Cardiovascular	Procedures requiring vascular anastomosis (e.g. Coronary Artery Bypass Grafting)	Creates anastomosis between two blood vessels	Lack of long-term outcomes data and patency rates
Magnetic navigation	everlinQ endoAVF System (Becton, Dickinson and Company, Franklin Lakes, NJ, USA)	Vascular	Alignment of blood vessels to allow for percutaneous arteriovenous fistula creation	End-stage renal disease requiring hemodialysis	Lack of long-term outcomes data and patency rates
Magnetic tracer	Sherlock 3CG Tip Confirmation System (Becton, Dickinson and Company, Franklin Lakes, NJ, USA)	Vascular	Need for central venous access	Active transmission of peripherally-inserted central venous catheter location during placement	Electromagnetic interference with implanted cardiac devices (e.g. ventricular assist device), Catheter malposition

## 2. Methods

For this narrative review, a literature search was conducted on PubMed, Scopus, and Web of Science for scientific articles published between 1,980 and 2,023 utilizing the following search terms: “magnet”, “magnetic force”, “surgery”, “musculoskeletal”, “spine”, “respiratory”, “airway”, “cardiovascular”, “cardiac”, “endovascular”, and “genitourinary”. Initial search identified 236 results which were screened for their utility in answering the following questions:.

(1) Does this device use magnetic force to treat a non-GI condition conventionally treated with surgical intervention?
(2) Does this device use one of the six core technical principles of magnetic surgery?
(3) Does this study report the use of this magnetic device in humans?

After adding these inclusion criteria and evaluating study credibility, the total number of texts included was narrowed to 78. Careful review of these included studies was then performed by the authors with a focus on the mechanism of action for each device, the reported benefit of magnetic force, results, and if available, data on complications and long-term outcomes with comparison to non-magnetosurgery techniques.

## 3. Magnetic surgery for musculoskeletal disorders

### 3.1. Spine and limb malformation/malalignment

Early-onset scoliosis is a deformity of the spine which can be congenital, idiopathic, or secondary to neuromuscular conditions such as cerebral palsy, muscular dystrophy, or spinal muscular atrophy (24). Scoliosis can negatively impact longitudinal growth and pulmonary function, as well as cause debilitating back pain. While bracing and physical therapy can aid patients affected with mild early-onset scoliosis, surgical intervention is often required for severe scoliosis. In the pediatric population, where continued longitudinal growth is a significant concern, dynamic growth rods—which fix the spine into an optimal growth vector while also allowing for continued growth—are ideal (24). However, repeated adjustment and lengthening of these dynamic growth rods requires multiple operations and increases exposure to general anesthesia.

**Figure 1:**
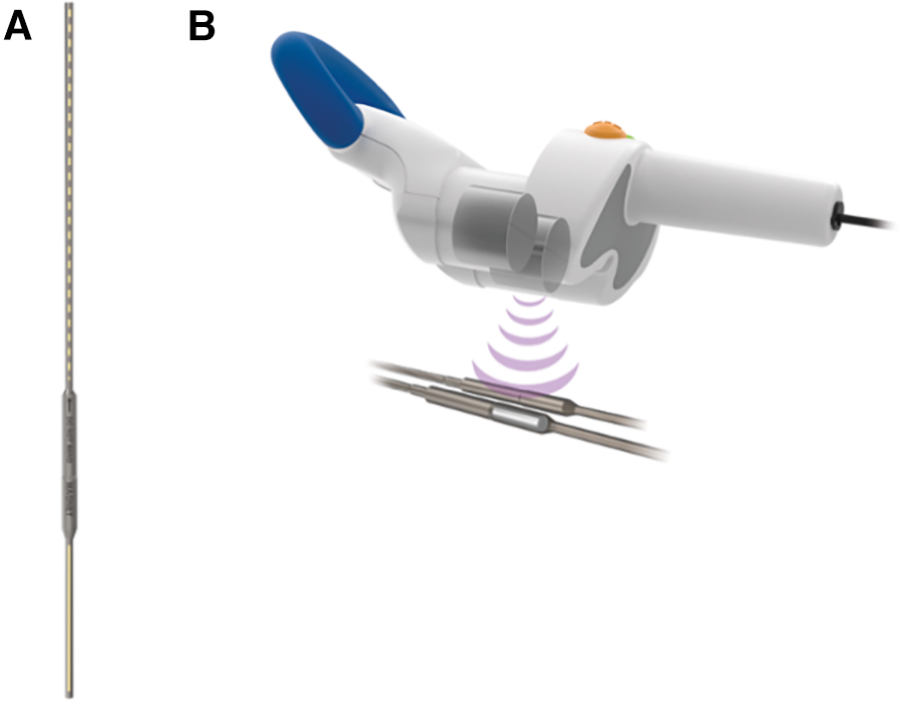
The MAGEC system (NuVasive Inc., San Diego, CA, USA) consists of an (**A**) implanted adjustable titanium rod internally fixated to the spine and (**B**) an external remote controller which acts on the internal rod's actuator to precisely lengthen or shorten the spinal growth rod without an additional surgery. (Permission for use granted by NuVasive Inc., San Diego, CA, USA).

Magnetic growth rods, such as the commercially available MAGEC System (NuVasive Inc., San Diego, CA, USA) utilize the magnetic driving technique to adjust rod length without the need for repeated operations under anesthesia (Table 1). While using a similar system of implanted titanium rods to fixate the spine, magnetic growth rods utilize an external magnetic device to drive a rotational force on the internal rod's actuator which is able to lengthen the rod precisely in a non-invasive fashion (Figure 1) (25). Magnetic growth rod systems have decreased the need for repeated operations, decreased anesthetic exposure, decreased infection rates, with non-inferior rates of implant failure compared to conventional dynamic growth rod systems (26–28). While novel technology is often limited by prohibitive cost, magnetic growth rod systems have also shown to decrease long-term cost after three years post-implantation which is likely due to the decrease in repeated operations (29). Due to the significant benefits of this novel use of the magnetic driving technique, magnetic growth rods have increased from <5% of all growth rod systems in 2007 up to 83% in 2016 (30). Thus, the introduction of magnetic force in the treatment of scoliosis has created an effective and less invasive alternative treatment for scoliosis in the pediatric population.

Similarly, limb length discrepancy secondary to fractures during active growth periods, tumor resections, congenital malformations, or congenital short stature often requires an orthopedic implant for treatment (31, 32). Longitudinal growth of the affected limb via osteogenesis is promoted through controlled limb distraction (33). This was initially performed with external fixation devices which were limited by pin-site infections, discomfort from pins/wires, and fractures secondary to external frame removal (34). Thus, these devices were developed into internalized (e.g., intramedullary nail lengthening), and eventually mechanically-driven motorized telescoping intramedullary nail systems (35). As internalized telescoping nail systems decreased patient discomfort, mitigated bone regenerate deformity, and facilitated an earlier return to full weight bearing (i.e., rehabilitation) compared to external fixation devices, magnetically-driven telescopic nails were explored and introduced in 2012 (36, 37). The PRECICE intramedullary limb lengthening system (NuVasive Inc., San Diego, CA, USA) is an example of a commercially-available telescoping intramedullary nail system that contains a rotating magnetically-controlled system for controlled nail extension or retraction (34, 37) (Table 1). The internal actuator is activated by an external remote controller containing two neodymium magnets which control the distraction rate (34, 37). In the pediatric population, this device has a reported mean lengthening range of 4.4–5.6 cm with nail accuracy (i.e., ratio of actual lengthening to planned lengthening) ranging from 91%–96% (38). While this device's use of the magnetic driving technique proves to be a feasible alternative to current motorized non-magnetic systems for the treatment of limb length discrepancy, limitations include device complications requiring surgical intervention (e.g., joint subluxation, delayed regenerate union, infection, nail fracture) and a wide variation in time to full weight bearing (range 21–132 days) (37–40).

### 3.2. Pectus Excavatum

Pectus excavatum is a congenital deformation of the connecting cartilages between the ribs and sternum which pushes the sternum posteriorly, leading to cardiopulmonary compression, which can decrease daily functionality and quality of life (41). Conventional repair methods include the modified Ravitch procedure which involves resection of the deformed cartilages, fracturing of the sternum, and internal fixation of the ribs/sternum, or the trans-mediastinal placement of titanium bars behind the sternum to gradually remodel the chest wall over a period of 1–2 years using the less invasive Nuss procedure (42, 43). Although effective, these procedures carry risks of injury to the heart, lungs, major blood vessels, and nerves controlling respiration (i.e., phrenic), as well as being associated with significant post-operative pain requiring prolonged opioid use and extended inpatient hospitalization (44). In addition, the Nuss procedure also carries the risk of internal bar rotation or malrotation requiring urgent re-operation (44).

**Figure 2:**
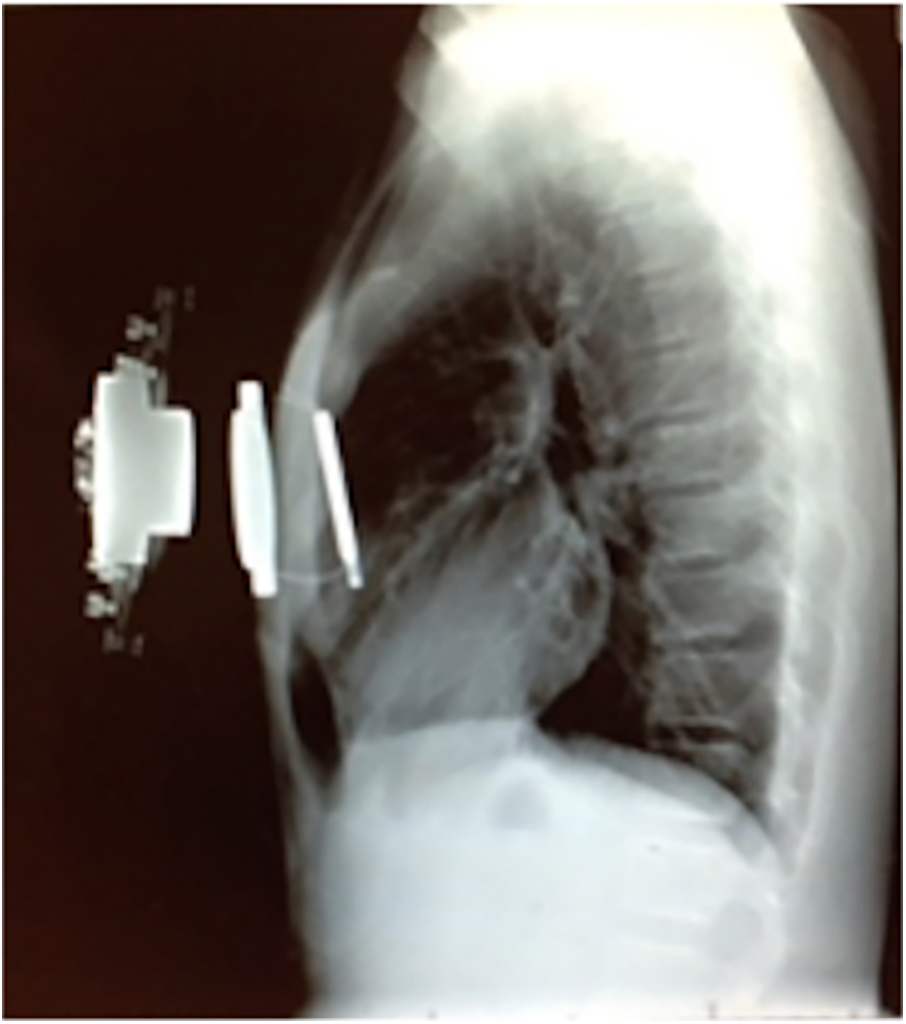
Lateral chest radiograph of a patient with pectus excavatum implanted with the magnimplant device and wearing the magnatract external brace. (Permission for use granted by Magnets-in-Me Inc., San Francisco, CA, USA).

**Figure 3:**
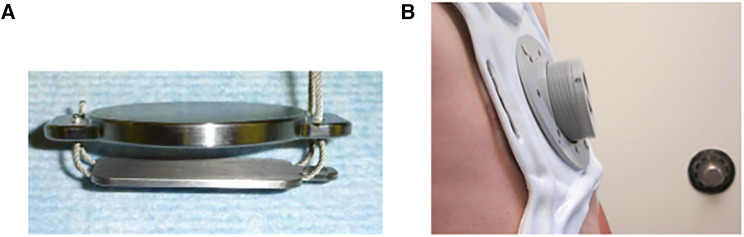
The magnetic mini-mover (3MP) device consists of: (**A**) magnimplant, a 2″ diameter titanium-sealed silicone steel disc containing a 1.5″ neodymium-iron-boron magnet with a ferromagnetic unidirectional focusing back plate, and (**B**) Magnatract, a custom fitted external orthotic brace containing a paired neodymium-iron-boron magnet with an integrated screw adjustor to calibrate the external force exerted on the Magnimplant. (Permission for use granted by Magnets in-Me Inc., San Francisco, CA, USA).

To mitigate these risks, the magnetic levitation technique has been utilized to create an alternative operative solution—the Magnetic Mini-Mover Procedure (3MP) (Table 1). 3MP uses a titanium-sealed neodymium-iron-boron magnet with a unidirectional ferromagnetic focusing plate—the Magnimplant (Hayes Manufacturing, Sunnyvale, CA, USA)—which is implanted to the sternum using titanium cables via a significantly less invasive operation (Figure 2) (45). Additionally, a custom orthotic external brace housing a paired magnet—the Magnatract (Hantel Technologies, Hayward, CA, USA)—exerts an external levitation force on the sternum to gradually remodel the chest wall (Figures 2 and 3) (45, 46). This approach offers several benefits, including decreased morbidity and risk of mortality, shorter operative times, decreased post-operative pain, and lower overall cost (42% less than the average cost for the Nuss or modified Ravitch procedure) (46, 47). In addition, an FDA-sponsored multi-center trial has demonstrated the ability of this technique to correct the pectus excavatum deformity and improve patient satisfaction up to 61% (46). However, early reports of device failure due to fatigue fracture of the titanium fixation cables have limited its adoption (46). Furthermore, the increased adoption of the Nuss procedure can be attributed to significant advances in post-operative analgesia techniques, such as intercostal nerve cryoablation, which have allowed for decreased opioid consumption and hospital length of stay (46, 48, 49). While use of the magnetic levitation technique has created a less invasive alternative to conventional pectus excavatum repair, further research is necessary to address these limitations and improve widespread adoption.

## 4. Magnetic surgery for respiratory disorders

### 4.1. Obstructive sleep apnea

**Figure 4:**
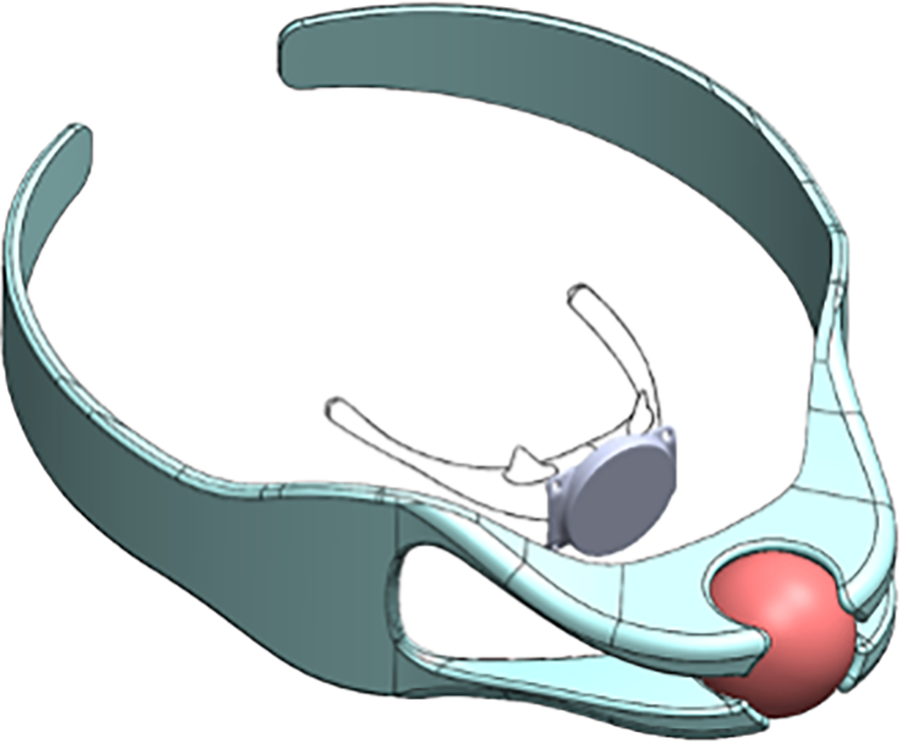
The magnetic apnea prevention (Mag-Nap) device uses a neodymiumiron-boron magnet encased in titanium with a ferromagnetic unidirectional back plate which is implanted to the hyoid bone (inner). The adjustable custom-fitted removable external neck brace (outer) contains a paired second magnet which exerts an external force to keep the airway open during sleep. (Permission for use granted by Mag-Nap Inc., San Francisco, CA).

Obstructive sleep apnea (OSA) is a common sleep disorder characterized by the partial or complete collapse of the upper airway during sleep, leading to a range of adverse effects, from daytime sleepiness to cardiovascular morbidity (50, 51). Multiple non-invasive and invasive therapies have been developed to treat OSA, but the most common approach is continuous positive airway pressure (CPAP) therapy. However, many patients struggle with CPAP adherence, necessitating alternative therapies that improve patient comfort and ease of use (52). The Magnetic Apnea Prevention (Mag-Nap) device (Mag-Nap Inc., UCSF Surgical Innovations, San Francisco, CA, USA) is being explored as an alternative OSA therapy which utilizes the magnetic levitation technique (Table 1). This device consists of an internal unidirectional magnet implanted to the hyoid bone via minimal superficial dissection and a paired external magnet housed within a customized orthotic neck brace (Figure 4). When the external neck brace is worn during sleep, the Mag-Nap device exerts an anterior force on the hyoid bone and underlying soft palate to minimize airway collapse during sleep. Preclinical studies in a cadaver model demonstrated that this device can exert the optimal force vector (perpendicular to neck contour) and necessary force (2 Newtons) to improve airway patency and airflow, compared those suboptimal force vectors achieved by alternative hyoid advancement techniques (53). Currently, a Phase I trial (NCT02431507) is actively recruiting patients in the United States with moderate-to-severe OSA (apnea-hypopnea index ≥15) and CPAP intolerance with the aim of evaluating the in-human safety and feasibility of this device (54). This use of magnetic levitation introduces a promising new therapeutic option for OSA that could improve patient comfort and adherence to therapy, compared to current therapies. However, completion of Phase I/II investigation is needed to fully evaluate the safety and efficacy of this device, as well as optimize its use for patients with OSA.

## 5. Magnetic surgery for cardiovascular disorders

### 5.1. Coronary artery stenosis

The magnetic compression technique, which has been commonly used in surgical devices for the GI tract (18, 55, 56), is now being explored by cardiac and vascular surgeons as a way to create vascular anastomoses (57–59). In patients with coronary artery stenosis leading to clinically significant myocardial ischemia, conventional revascularization via bypass grafting to a separate artery requires prolonged clamping of the artery to allow for a handsewn anastomosis. This technique can be time-consuming and carries the risk of complications secondary to prolonged ischemia.

To address this issue, magnetic vascular ports have been developed. These permanent magnetic vascular port devices are deployed within two separate arteries and are coupled to form an instant anastomosis (60) (Table 1). The Magnetic Vascular Positioner Series 6,000 Distal Anastomosis System (Ventrica Inc., Fremont, CA, USA) has been used in the Minimally Invasive Direct Coronary Artery Bypass (MIDCAB) procedure to anastomose the left internal mammary artery to left anterior descending coronary artery, resulting in decreased anastomosis time (mean 199 s) and decreased total operative time (mean 128 min), without device-related adverse events or decrease in patency rates at 6-month follow-up (60–62). This device has also been explored to repair injuries to the left internal mammary artery (63), and further research is actively exploring the potential of this magnetic compression technique in preclinical models to create arteriovenous and veno-venous anastomoses (64–66). However, widespread adoption is limited by the lack of published long-term patency data with this device/technique. As this technology continues to advance, it may offer an innovative solution for patients with life- or limb-threatening ischemia by reducing operative time, and subsequently reducing ischemia time.

### 5.2. Endovascular procedures

Similar to the adoption of magnetic compression techniques from GI surgery to cardiovascular surgery, magnetic tracer techniques which have been developed in the placement of nasoenteric feeding tubes are now being employed in the development of novel endovascular devices (15, 67). Peripherally inserted central venous catheters (i.e., PICC lines) are commonly deployed in adult and pediatric patients for the administration of medications or parenteral nutrition. Conventional guidance of these intravascular catheters during insertion often requires elevated levels of radiation exposure (e.g., repeated radiographs, continuous fluoroscopy) to the patient and provider in order to minimize the risk of catheter malposition or vascular injury. The Sherlock 3CG Tip Confirmation system (Becton, Dickinson, and Company, Franklin Lakes, NJ, USA) utilizes the magnetic tracing technique to provide real-time tracking of the intravascular magnetic catheter tip with an external sensor on the patient's sternum (67). Use of magnetic tracing has demonstrated reliability by decreasing rates of catheter malposition, while also improving patient/provider safety by decreasing procedure time and radiation exposure (67, 68).

Magnetic tracer technology is also being explored to create a novel minimally-invasive endovascular technique for arteriovenous fistula (AVF) creation in patients requiring long-term hemodialysis (69). Conventional AVF creation requires an open dissection of the target artery and vein, prolonged clamping of both vessels, and a handsewn anastomosis. In contrast, endovascular AVF creation with the EverlinQ EndoAVF System (Becton, Dickinson, and Company, Franklin Lakes, NJ, USA) uses the magnetic tracing technique to navigate intravascular catheters in the artery and vein to their target positions, followed by magnetic coupling to align and oppose the artery and vein for radiofrequency welding of the vessel walls (59, 69). Thus, forming a new AVF via a minimally invasive percutaneous approach. These examples demonstrate how the magnetic tracer technique is being utilized to navigate intravascular catheters in existing and novel endovascular procedures.

## 6. Magnetic surgery for genitourinary disorders

### 6.1. Ureteral stent retrieval

Ureteral stents are commonly used for a variety of indications including relief of ureteral obstructions (e.g., nephrolithiasis), during ureteral anastomoses (e.g., renal transplant, ureter injury), or to assist in ureter localization during pelvic or retroperitoneal operations (70). However, removal of these stents typically requires cystoscopy, which can be associated with significant pain, prolonged procedure time, and may even require the use of sedative medications (71, 72). In order to improve patient-reported outcomes, the magnetic anchoring technique has been adopted to develop a paired magnetic device for bedside ureteral stent removal. The Magnetic Blackstar device (Urovision-Urotec, Achenmuhle, Germany) is a novel commercially available magnetic double-J stent that utilizes an inner magnetic anchor located at the distal end of the ureteral stent and a paired magnetic anchor on a stent retrieval device. The retrieval device is introduced into the bladder and anchors to the internal magnet to extract the stent without the need for cystoscopy (73). This procedure can be performed at the patient's bedside and has been shown to be highly effective in removing ureteral stents with reduced pain, shorter procedure times, and lower costs, compared to conventional stent removal with cystoscopy (71, 74, 75). Additionally, the device has been shown to be feasible for stent removal without general anesthesia in the pediatric population (72). Thus, the magnetic double-J stent is a novel device that has utilized the magnetic anchoring technique to create a novel technique for ureteral stent extraction without cystoscopy. This device has great potential for improving patient comfort, decreasing healthcare costs, and reducing anesthetic exposure.

## 7. Considerations during magnetic surgical device development

As the adoption of magnetic surgical devices to treat non-GI disorders has grown in recent years, careful considerations during the development and early implementation phases is prudent to ensure their safety and efficacy. One of the key considerations during the development of magnetic surgical devices is the coupling strength between paired magnets. Coupling strength decreases exponentially with increasing distance between paired magnets, which makes it a limiting factor in the magnetic levitation technique (76). Understanding of the heterogeneity in subcutaneous tissue thickness, and subsequent magnetic coupling strength, is vital for the proper design and allocation of paired magnets. The thickness of subcutaneous tissue also has significant implications for proper patient selection and the consideration of pre-implantation anatomic augmentation to optimize device efficacy.

Another critical aspect of magnetic surgical device development for prolonged (i.e., permanent, semi-permanent) implantation is the exposure risk of a sustained static magnetic field. For permanently implanted devices (e.g., vascular ports) or semi-permanent devices (e.g., MAGEC system, Mag-Nap, 3MP), the maximum safe static magnetic field exposure is 400 mT (77). Additionally, these devices risk altering the function of other implanted devices, such as cardiac pacemakers or implantable cardioverter-defibrillators, making it essential to ensure that the static magnetic field exposure to these devices is below 0.5 mT (77). However, as the force generated by magnetic devices is inversely related to distance, magnetic devices that generate force greater than 0.5 mT may be used if they are implanted at a sufficient distance away from the existing implanted device (78).

As with magnetic surgical devices used in the GI tract, prior to device implantation, one must also balance the benefit of the permanent/semi-permanent implanted device with the risk of not being able to undergo an MRI in the future. Due to the strength of the magnetic field created by an MRI, the risk of MRI exposure to an implanted magnetic device could be catastrophic and/or fatal. Therefore, it is vital to consider the implications of device implantation on future MRI usage.

Lastly, early adoption and implementation of magnetic surgical devices for non-GI disorders requires careful evaluation of patient-reported outcomes, complication rates, and long-term outcomes (i.e., durability of treatment and device). As the current body of literature surrounding magnetic surgical devices for non-GI disorders is vast and skewed toward musculoskeletal disorders, the majority of prior studies focus on the feasibility of device use and short-term outcomes. Thus, long-term follow up data and prospective comparison of magnetic devices to non-magnetic devices are areas of opportunity for continued study in this field.

## 8. Conclusion

The use of magnetic surgical devices in organ systems beyond the GI tract has evolved rapidly over the past few decades. Although the vast experience with magnetic force has been primarily in the GI tract, clinical applications across various organ systems have demonstrated the effectiveness of established magnetic surgical principles. These shared principles can be applied to treat a wide range of pathologies including musculoskeletal, respiratory, cardiovascular, and genitourinary disorders. As this is a rapidly evolving field, further innovation, research, and technological advancements are expected to continue expanding the use of magnetic surgical devices beyond the GI tract—improving patient outcomes and revolutionizing minimally-invasive surgery.
